# Anomalous anti-damping in sputtered *β*-Ta/Py bilayer system

**DOI:** 10.1038/srep19488

**Published:** 2016-01-19

**Authors:** Nilamani Behera, Sujeet Chaudhary, Dinesh K. Pandya

**Affiliations:** 1 Thin Film Laboratory, Department of Physics, Indian Institute of Technology Delhi, New Delhi 110016, INDIA

## Abstract

Anomalous decrease in effective damping parameter *α*_*eff*_ in sputtered Ni_81_Fe_19_ (Py) thin films in contact with a very thin *β*-Ta layer without necessitating the flow of DC-current is observed. This reduction in *α*_*eff*_, which is also referred to as *anti-damping* effect, is found to be critically dependent on the thickness of *β*-Ta layer; *α*_*eff*_ being highest, i.e., 0.0093 ± 0.0003 for bare Ni_81_Fe_19_(18 nm)/SiO_2_/Si compared to the smallest value of 0.0077 ± 0.0001 for *β-*Ta(6 nm)/Py(18 nm)/SiO_2_/Si. This anomalous anti-damping effect is understood in terms of interfacial Rashba effect associated with the formation of a thin protective Ta_2_O_5_ barrier layer and also the spin pumping induced non-equilibrium diffusive spin-accumulation effect in *β-*Ta layer near the Ta/Py interface which induces additional spin orbit torque (SOT) on the moments in Py leading to reduction in 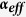. The fitting of 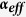 (*t*_*Ta*_) revealed an anomalous negative interfacial spin mixing conductance, 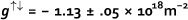
and spin diffusion length,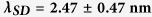. The increase in *α*_*eff*_ observed above *t*_*Ta*_ = 6 nm is attributed to *t*he weakening of SOT at higher *t*_*Ta*_. The study highlights the potential of employing *β-*Ta based nanostructures in developing low power spintronic devices having tunable as well as low value of *α.*

In recent years, the Rashba spin orbit interaction (RSOI) has emerged as a powerful tool for significantly enhancing the spin transfer torque (STT) in ferromagnetic (FM) layer when it is in contact with the heavy metallic nonmagnetic (NM) layer, i.e., in NM/FM hetero-structures, e.g., Bi_2_Se_3_/Py, Ta/CoFeB/MgO, etc^1^,^2^,^3^,^4^,^5^,^6^,^7^,^8^. In the presence of charge current through the NM layer, the RSOI forces the spins at the interface via spin Hall effect (SHE) in transverse direction thereby creating a non equilibrium spin accumulation near the NM/FM interface^2^. In presence of a dc-magnetic field, the accumulated interfacial non-equilibrium spin density interacts with the magnetization of FM layer via ferromagnetic exchange coupling and eventually reverses the magnetization at high current density. Referred to as the anti-damping of the magnetization precession^3^,^4^,^9^,^10^,^11^,^12^,^13^,^14^, this phenomenon essentially lowers the Gilbert’s damping constant (*α*), when compared to the case of bare FM. Similar RSOI like anti-damping effect also originates from the Berry curvature, associated with the phase with broken inversion symmetry, which produces SOT that counteracts the magnetization dynamics^15^. As the effect, by its fundamental origin, relies on the local accumulation of spins near the interface, the Rashba effect is also referred to as *interfacial* spin Hall effect^3^. The anti-damping observed in Rashba effect fundamentally arises due to local modification in the spin orbit interaction near the interface, which gives rise to the Rashba spin orbit torque (RSOT) necessary for lowering of *α*. It may be pointed out that this so-called SHE-RSOT, which is significant only when thickness of NM layer 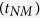 is comparable/smaller than its spin-diffusion length 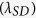, is quite different from the bulk SHE driven STT (observed when *t*_*NM*_ > *λ*_*SD*_) wherein both damping and anti-damping effects could occur depending upon the magnitude and direction of DC-current^16^. In this later case of bulk SHE-STT in NM/FM bilayers, interfacial contribution to STT arising due to local interactions at the interface are usually very weak and hence are often ignored^3^. Now a days, physics related to interface is playing an important role in technological applications like magnetic random access-memory, magnetic data storage and spin based logic devices. Hence, the influence of the nature of interface in NM/FM bilayer system on the spin pumping is of paramount importance for realization of spintronic devices.

Recently theoretical groups have reported that the RSOI also occurs in ferromagnetic semiconductors (FMS), e.g., Mn_x_Ga_1−x_As, etc^13^,^14^,^15^,^17^,^18^. It is to be noted that in all these reports, the non-equilibrium spin density is created when the FMS is subjected to an *rf*-current. This non-equilibrium spin density exerts SOT on the magnetization via the exchange coupling between the carrier’s magnetic moment. However, at higher currents, the noise due to both the Oersted field and the associated heating effects dominates over SOT and leads to suppression or disappearance of anti-damping^19^,^20^,^21^,^22^,^23^. This makes it very difficult to separate the contributions from the RSOT and SHE-STT.

In this communication, we present the experimental evidence of anti-damping SOT in a *β*-Ta/Py/SiO_2_/Si(100) bilayers without any DC-current flowing through Ta. Although the observed anti-damping effect in these *β*-Ta/Py bilayers bears similarity with regard to the DC- current induced anti-damping effect observed in Ta/CoFeB bilayer systems^24^,^25^,^26^, the anti-damping observed in the present case of *β*-Ta/Py bilayers is, however, anomalous since it is observed in the absence of DC-current to *β*-Ta layer. Based on the analyses of the line boarding in the ferromagnetic resonance (FMR) spectra recorded on the bilayers having different Ta layer thickness (*t*_*Ta*_) deposited *in-situ* over the Py layer of constant thickness *t*_*Py*_ = 18 nm, it is proposed that the observed anti-damping effect has its origin associated with a Rashba like interfacial SOT arising due to the spin accumulation at the *β*-Ta/Py interface^27^,^28^,^29^,^30^,^31^. The anti-damping effect is found to be systematically dependent on *t*_*Ta*_; becoming more and more pronounced with the increase in 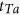 till about 6 nm. Above *t*_*Ta*_ ~ 6 nm, its strength decreases monotonically and becomes more or less independent of 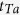 above 8 nm. These experimental results which are manifestations of the FMR induced spin-pumping mechanism in *β*-Ta/Py bilayers are explained in terms of negative interfacial effective spin mixing conductance (*g*^↑↓^) which depends on 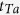. The studies suggest that Ta can act as a potential candidate material for inducing Rashba like-torque leading to lower *α*, which could be useful for developing potential spintronics devices with relatively low power dissipation due to the absence of any DC-current.

Py thin films (thickness 18 nm) were grown at room temperature on SiO_2_/(100)Si substrates (SiO_2_ is the native oxide layer on Si) by pulsed DC magnetron sputtering using 99.99% pure Py target. The *β*-Ta layers of different thicknesses varying from 1–24 nm (in steps of 1 nm from 1 to 8, 2 nm from 8 to 12 and 4 nm from 12–24 nm) were grown on top of Py(18 nm) by using 99.99% pure Ta target. The base pressure of sputtering chamber was ∼2 × 10^−7^ Torr and Ar working pressure of ~3.2 × 10^−3^ Torr was maintained during bilayer growth. The in-plane magnetization of *β-*Ta/Py thin films was measured by Physical Property Measurement System (PPMS) (Model *Evercool-II* from *Quantum Design Inc*) facility at IIT Delhi. The X-Ray diffraction studies on these thin films have been done by using *X’Pert-Pro* x-ray diffractometer (XRD) with Cu-K_α_ (1.54 Å) source for studying the phase purity and orientation aspects of the Ta and Py thin films. The thicknesses of individual layers and interface roughness (~0.4 nm) were accurately determined by x-ray reflectivity (XRR) measurements. The in-plane resonance field 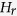 and linewidth 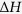 were measured by using broadband lock-in-amplifier based ferromagnetic resonance (LIA-FMR) technique developed in-house with the help of a vector network analyzer (VNA) in an in-plane magnetic field configuration employing a coplanar waveguide (CPW). Figure 1a shows the schematic of the FMR set up. The VNA (*HP* make Model *8719 ES*) sourced the microwave signal at a particular frequency to the CPW placed within in the pole gap of an electromagnet as shown in Fig. 1a. The FM/NM bilayer thin film sample (size *1* × *4 *mm^2^) is mounted on the central signal line (*S*) of CPW (see Fig. 1b) such that the film-side is in contact with *S* while the substrate side faces upward. In this geometry, the external DC-magnetic field *H* from the electromagnet and microwave field *h*_*rf*_ of the CPW are transverse to each other, and both lie parallel to the film-plane. The resonance condition is obtained by sweeping *H* at different constant values of microwave frequency (5–10 GHz). To improve the signal to noise ratio, the DC-magnetic field was modulated using an AC-field of an optimized strength of 1.3 Oe at 211.5 Hz frequency which was obtained by powering a pair of Helmholtz coils from the reference oscillator of the lock-in-amplifier from *Stanford Research Systems Inc*. (Model–*SR 830 DSP*). The output signal, essentially the derivative of the signal from the sample, locked at 211.5 Hz was detected by the LIA via an RF-diode detector. X-ray photoelectron spectroscopic (XPS) spectra were recorded using *SPECS* make system which uses MgK_α_ (1253.6 eV) source and hemispherical energy analyzer (pass energy of 40 eV with a resolution of ~0.3 eV) to probe the surface of the *β*-Ta/Py bilayers.

Figure 2 shows the X-Ray diffraction patterns recorded in *θ*–*2θ* mode on Py (18 nm), *β*-Ta (30 nm) and *β*-Ta 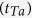/Py bilayer thin films, where 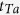 corresponds to thickness of Ta layer. The formation of highly textured *β*-Ta phase is established from (i) the presence of very intense (*002)* and *(004)* peaks from the *β*-Ta phase at 2θ value of 33.5° and 70.1°, respectively, and (ii) the absence of the most intense peaks of *α*-Ta phase, namely the *(110*) peak at 2θ = 38.4° (which overlaps with *(202)* and *(211)* of *β*-Ta) and the isolated *(200)* peak at 2θ = 56.0°. It may be noted that, in the present case, the formation of the phase pure *β*-Ta required a relatively higher sputtering power ~150 W (over 2” dia target area). The 2θ peak position of 33.2° corresponding to *d* value of 2.70 Å (very close to reported value of 2.67 Å for Ta)^32^ reveals that the growth of Ta thin films is in desired tetragonal *β*-phase having preferential orientation of the *(200)* planes. This is consistent with its measured value of resistivity of 180 μΩ.cm at room temperature which agreed excellently well with the reported values in literature^24^,^33^. While we did not observe any discernible XRD peak in the bare Py film in *θ–2θ* scan mode due to its small thickness (18 nm), the glancing angle XRD (inset of Fig. 2) pattern recorded at 0.5 and 1°, however, confirmed the growth of Py having (111) preferred orientation.

The spin dynamic response of these bilayer films is investigated by analyzing the FMR spectra recorded by reducing the external dc-magnetic field from the saturation magnetization state of *β*-Ta/Py bilayers at different constant microwave frequencies lying in the range of 4–10 GHz. For determining the 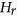 and 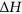 at constant frequencies, the observed FMR spectra were fitted with the derivative of Lorentzian function as shown by solid lines (Fig. 3(a)). The frequency dependence of 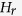
observed for *β*-Ta(1 nm)/Py (18 nm) bilayer films is shown in Fig. 3(b). The observed 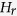 vs. *f* data are fitted (solid lines in Fig. 3(b)) by using the Kittel’s equation^34^


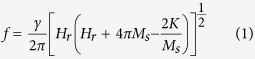


where *γ* is the gyromagnetic ratio 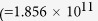 *Hz/T*) when the spectroscopic splitting factor *g* is taken as 2.1. Given the higher thickness of Py layer, i.e., 18 nm in the present case, it is very reasonable to ignore the anisotropy term ‘*−2K/M*_*s*_’ in equation (1), and hence the Kittel’s equation reduces in the present case to,


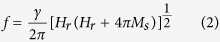


The values of saturation magnetization 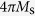, obtained from the fitting of *H*_*r*_ versus *f* data of the bilayers, are found to lie in the range of 938–1013 mT. Within the error of estimation, the 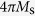 values so determined on *β*-Ta(*t*_*Ta*_)/Py bilayers are clearly large compared to that of bare Py layer (see dotted line in Fig. 4), clearly ruling out the presence of any magnetically dead-layer in these bilayers having different 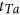. This is in sharp contrast to the reported work on Ta/CoFeB^33^,^35^. Instead, in the present case, an increase in 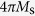 value with increase in 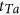 till about 6 nm can be noted from Fig. 4. It is conjectured that this increase in 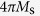 (~8.0% higher compared to that in bare Py layer) inferred from the FMR measurement could result from the presence of the extra spin density due to diffusive spin accumulation^3^,^36^ in *β*-Ta layer. This accumulation, which has induced extra magnetization, is indeed theoretically shown to be originating via the local strong spin orbit coupling near the interface due to the proximity with the FM layer^1^,^3^,^13^,^36^. There exist some reports in literature wherein the magnetic proximity effect is reported in Ta/FM structures^37^,^38^.The 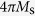 value as determined from the PPMS measurement of the *β*-Ta(6 nm)/Py(18 nm) bilayer was found to be 805.24 emu/cc (≈1012 mT), which agrees reasonably well with the value estimated from the fitting of FMR data on the same sample (Fig. 4).

In order to have a deeper insight of the FMR induced spin pumping in these *β*-Ta/Py bilayers, we now turn to the frequency dependence of 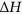. Figure 5 shows the observed frequency dependence of 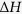 (open data symbols) for these *β*-Ta (*t*_*Ta*_)/Py bilayer thin films. It can be seen that 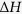 increases linearly with the resonance frequency. This linear increase clearly suggests that the damping of the precession in this *β*-Ta/Py bilayer system is governed by the intrinsic Gilbert’s phenomena, i.e., magnon-electron (ME) scattering. The observed frequency dependence of 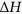 is fitted with the equation^39^,


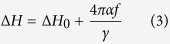


where, 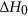 accounts for line-broadening owing to the extrinsic contributions (e.g., scattering due to magnetic inhomogeneities, etc.) to Gilbert’s damping. Normally the presence of the inhomogeneous broadening contribution 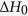 is indicative of the inferior film-quality. In the present case, ΔΗ_0_ ~ 0 (0 to 0.2 mT, i.e., only 1–5% of ΔΗ, see Figs. 5 and 6(a)) for various bilayers, indicating the excellent film quality of these samples. The 2^nd^ term in equation (3) represents the intrinsic ME contribution to the line-width and is proportional to the Gilbert’s damping constant. In fact, for bilayers such as *β*-Ta/Py in the present case, the usual damping parameter *α* should be replaced with 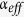 so as to account for the extra contribution 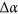 coming from the spin pumping contributions, i.e., effective Gilbert’s damping constant, 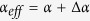. From the fittings of 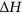 versus *f* data of Fig. 5, we obtained the variation in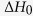 and 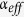 as 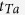
is varied in 1–24 nm range. The results are plotted in Fig. 6(a,b), respectively. Within the error of fitting, the inhomogeneous line broadening 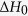 can be seen to be nearly constant at ~0.2 mT for different thicknesses of Ta. It may be noted from Fig. 6(b) that irrespective of the values of 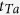, the observed values of 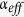 for these *β*-Ta(*t*_Ta_)/Py bilayers are smaller than that of the bare Py sample which possessed *α* = 0.0093 ± 0.0003. This observed decrease in 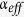 is quite remarkable and suggests the existence of anti-damping effect (even in the absence of any DC-current) in these *β*-Ta(*t*_Ta_)/Py bilayers. In addition, 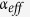
initially exhibits a monotonic decrease as 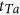 is increased, and attains the smallest value of 0.0077 ± 0.0001 near 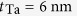 (Fig. 6(b)). Thereafter, 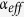 exhibited a relatively sharp increase to 0.0086 ± 0.0001 at *t*_*Ta*_ = 8 nm followed by a relatively small variation in *α*_*eff*_.

As 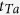 is increased from 0 to 6 nm, the observed significant anti-damping (i.e., decrease in the effective *α* and quantified by Δ*α* = −0.0016) in these *β*-Ta/Py samples can be understood on the basis of generation of (Rashba like) interfacial SOT at the interface of *β*-Ta/Py bilayers. It is emphasized here that the origin of anti-damping effect observed in the present case cannot be accounted due to the SHE-STT, since the decrease in 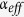 is observed in the absence of DC- current. Combined with the observed increase in 4*πM*_s_ due to the proximity induced strong spin-orbit coupling (Fig. 4), the FMR results (Fig. 6b) therefore substantiate the presence of strong SOT leading to negative 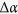 (anti-damping) when Ta is deposited on Py layer.

To have further deeper insight about the anti-damping effect observed in the present case, the present results have been analyzed within the framework of a theoretical model proposed by Tserskovnyak/Bauer *et al.*^27^,^28^,^29^,^30^,^31^ on the basis of diffusive spin accumulation hypothesis. According to this model, it was shown that in the absence of DC-current in a NM/FM bilayer system, the spin pumping into the NM layer^40^ is usually governed by the interfacial spin mixing conductance (*g*^↑↓^)^41^,^42^, which basically indicates the efficiency of the transfer of spin angular momentum 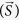 from FM to NM layer. The spin current density 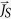 induced as a result of spin pumping is expressed as:


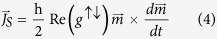


where Re(*g*^↑↓^) is the real-part of the *g*^↑↓^. It may be noted that 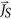 is polarized perpendicular both to the instantaneous magnetization 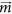 and to its time derivative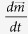. Before we further discuss 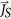, we recall that this transfer of 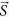 from FM to NM layer is known^27^ to depend critically upon the nature of the NM layer via a parameter *∈*, which is defined as the ratio of spin flip parameter (*τ*_*sf*_) within the NM layer to the spin-injection/pumping rate (*τ*_*sp*_) from the FM layer. Tserskovnyak/Bauer *et al.*^27^,^28^,^29^,^30^,^31^ showed that in the case of *∈* > 0.1, the spins accumulation at the interface is not possible in sharp contrast to the case when *∈* < 0.1^43^,^44^. In the later case, the spin angular momentum 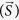 associated with the spins accumulated at the FM/NM interface creates a non-equilibrium spin density in the NM layer^27^,^31^. As a consequence of this, a back flow of spin current (indicated by 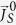) into the FM layer takes place. It was further theoretically established that provided the NM layer possesses adequate spin orbit coupling (SOC), then during this back flow, the component of 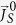 parallel to the instantaneous magnetization 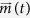 of FM layer counteracts the spin pumping from FM layer, thereby effectively suppresses the spin pumping into NM. On the other hand, the transverse component of 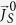 generates an additional torque (SOT) on the in-plane 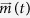 of FM layer. The magnitude of such SOT is reported to scale with 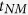 till about 2*λ*_*SD*_^2^,^36^. In the present case of *β*-Ta (*t*_Ta_)/Py(18 nm) bilayers, existence of this extra torque (i.e., SOT) could account for the observed decrease in 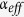 (or the anti-damping effect) since *∈* < 0.1 for the nonmagnetic Ta layer present in our bilayers^43^,^44^,^45^.

In order to ascertain the presence of SOT (without DC-current), the observed thickness dependence of 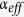 in these *β*-Ta(*t*_*Ta*_)/Py(18 nm) bilayers (Fig. 6(b)) were quantitatively analyzed using the theoretical predictions of decrease in interfacial diffusive spin accumulation with the thickness of NM layer^27^,^28^,^29^,^30^,^31^. The spin accumulation at the interface is very sensitive to spin diffusion length 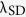 of *β-*Ta layer^33^,^36^. We argue that for the thickness regime, 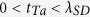
* (≈ 2.74 nm for β-*Ta)^25^,^36^, the strength of the spin accumulation near the interface regime is expected to dominate over the effect of SOC of the *β-*Ta layer. This suggests that instead of damping, the spin-accumulation can generate 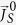 which can contribute to the smaller *α*. Understandably, there would be a case corresponding to a critical value of 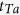 above which the SOT caused by 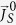 weakens (due to lowering of 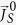 as 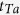 is increased) compared to that when spin accumulation is absent, which would result in increase in 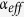
36. In the present case of *β-*Ta, this crossover in 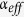 is expected to lie near *t*_*Ta*_ = 6 nm (which is ~2l_SD_, since above this Ta film-thickness, no back flow of 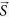 to FM layer occurs due to the loss of the spin coherence within the bulk of Ta^43^). The initial decrease in 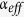 observed with the increase in 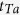, therefore, finds a natural explanation within this diffusive spin accumulation model. Above *t*_*Ta*_ = 6 nm, the 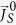 diminishes due to the decrease in spin accumulation expected at higher 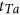, accounting for the rise in 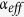 observed above *t*_*Ta*_ = 6 nm. These results are in excellent agreement with the results obtained by Jiao *et al.* who observed higher ISHE signal in different bilayers of Py with Ta, Pt, and Pd, only in thickness regime *t*_*NM*_ ≤ *λ*_*SD*_ (ref. 36).

According to ref. 46, in the presence of spin accumulation in *β*-Ta the net change in effective Gilbert’s damping due to SOT is determined in terms of *g*^↑↓^,


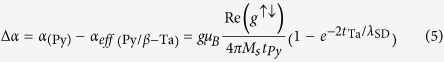


where *g* = 2.1, 
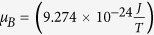
, is the Bohr magneton. This equation shows that SOT is an interfacial effect and hence is expected to decrease with the thickness of Ta layer beyond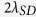. Figure 7 shows the plot of 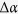 versus 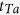. From the fit of the data in Fig. 7 to equation (5), one can experimentally find the interfacial spin mixing conductance and the spin diffusion length of Ta layer. The value of *g*^↑↓^ and 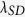 determined from the fitting are 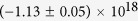 m^−2^ and 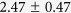 nm, respectively. This value of 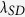 is very close to the theoretical reported value 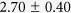 nm by Morota *et al.*^25^ We can also determine the transparency (*T*) of interface (which accounts for the flow of spin current density that diffuses from FM layer to the NM layer and the actual spin current density generated via spin pumping process from FM layer; such that *T* < 1) by using^47^


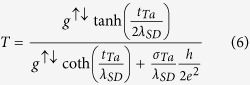


Here *g*^↑↓^ and *λ*_*SD*_ are determined from the fitting parameters of equation (5), *σ*_*Ta*_ is the conductivity of *β*-Ta layer ( = 5.5 × 10^5 ^ohm^−1^ m^−1^), *h* is the Planck’s constant, and *e* is the charge of the electron. The calculated value of *T* from equation (6) is −0.98(±0.05) at *t*_*Ta*_ = 6 nm. The negative and higher value of *T* combined with low and negative value of *g*^↑↓^ in *β*-Ta/Py bilayer suggests that there is poor band matching between Py and *β*-Ta layers that causes back reflection of 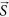 into the FM layer from the interface. This leads to the possibility of exchange of torque by the spins present on the two sides in close proximity to the NM/FM interface by Rashba effect^8^,^13^,^14^,^26^,^47^,^48^,^49^. Thus, it is concluded that the observed anomalous anti-damping in *β*-Ta/Py bilayers could be accounted for by the presence of non-equilibrium spin density (providing strong experimental support to the Tserskovnyak’s theoretical model) near the NM/FM interface. It is reiterated that anti-damping is also reported earlier in ferromagnetic semiconductors like (Mn_x_Ga_1−x_As)^15^ and at the interface of NM/FM bilayer system^33^ due to generation of RSOT by non-equilibrium spin density with the application of RF and dc-currents.

While the anomalous anti-damping observed in these *β-*Ta(1–24 nm)/Py(18 nm) bilayers appears to provide the strong experimental support in favor of the theoretical predictions of decrease in interfacial diffusive spin accumulation with the thickness of NM layer^27^,^28^,^29^,^30^,^31^, it is quite likely that the decrease in 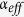 in Ta capped Py layers, compared to higher 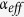 of bare Py, could also be a result of the protection of the underlying Py layer by the formation of protective oxide barrier. To ascertain the contribution of oxide barrier in lowering the 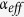, we performed XPS measurements and XRR simulations on a few representative samples, namely the bare Py film and Py/*β*-Ta(4 nm) bilayer. The XPS spectrum recorded on bare Py film (see Fig. 8(a)) did not clearly support the formation of (antiferromagnetic) NiO. On the other hand, the XPS spectra of Py/*β*-Ta(4 nm) bilayer clearly revealed the formation of a thin Ta_2_O_5_ top layer protecting the Ta as well as Py under layer (see Fig. 8b,c for Ta-4f and O-1s levels, respectively)^50^. Thus, the bilayer with *t*_*Ta*_ = 1 nm is, in fact, Ta_2_O_5_/Py. This finds support from ref. 16 wherein 1 nm Ta cap is reported to fully protect the surface of the Py layer from its oxidation. Since Ta_2_O_5_ is known to possess inversion asymmetry [ref. 51], this bilayer is expected to exhibit significant amount of anti-damping SOT^51^ due to the non-equilibrium spin-accumulation arising predominantly because of interfacial Rashba effect, consistent with the profound drop observed in *α*_*eff*_ in the *t*_*Ta*_ = 1 nm bilayer (Fig. 6b) as compared to the bare Py. A similar fall in *α*_*eff*_ was also reported by Allen *et al.* (ref. 33). It is evident that at higher *t*_*Ta*_, the thin metallic layer of Ta will eventually isolate the Py from Ta_2_O_5_. Figures 9a–c show the simulated and experimental XRR spectra recorded on samples with *t*_*Ta*_ = 0, 3 and 4 nm by considering an oxide layer on the top of the bilayers. The fitted value of layer thickness and its roughness together match quite well with the nominal thicknesses of the Ta and Py layers. It is evident that XRR simulations provide the additional experimental support in favor of a thin protective cap of NiO (*t*~1 nm) in Py and of Ta_2_O_5_ (~2 nm) on top of *β*-Ta(3,4 nm)/Py(18 nm) bilayers. Thus, the XPS and XRR measurements together suggest that the anti-damping effect in *β*-Ta(*t*_*Ta*_)/Py(18 nm) bilayers occurs due to the interfacial Rashba effect (predominant till *t*_*Ta*_~3 nm) and to the spin pumping induced spin accumulation in *β-*Ta layer below *t*_*Ta*_ < 6 nm. Eventually, when *t*_*Ta*_ is increased above ~6 nm (≅2*λ*_*SD*_), *α*_*eff*_ understandably starts exhibiting usual spin-pumping driven damping effect due to the transfer of spin angular momentum in *β-*Ta. It is to be noted here that recently Akylo *et al.*^51^, Kim *et al.*^52^, and Qiu *et al*.^53^ have also independently established the enhancement in ‘effective field’ due to Rashba effect with the increase in *t*_*NM*_ having strong spin orbit coupling.

In summary, the FMR studies performed on *β-*Ta(1–24 nm)/Py(18 nm)/SiO_2_/Si revealed an anomalous decrease in the effective Gilbert’s damping constant as compared to the bare Py(18 nm) layer. The analyses of the FMR line broadening data suggests that the anomalous behavior could be satisfactorily understood by considering the dominance of Rasbha like spin orbit torque at the interface of *β-*Ta/Py bilayer due to formation of a thin protective Ta_2_O_5_ barrier layer and the spin pumping induced non-equilibrium diffusive spin-accumulation effect in *β-*Ta layer until its thickness is smaller than its spin diffusion length, i.e., *t*_*Ta*_ ≤ 6 nm. The study clearly establishes that owing to very small spin diffusion length, the thickness of the non-magnetic *β*-Ta layer in these bilayers is very critical to the Gilbert’s damping in the adjacent Py layer. Above 6 nm thickness of Ta, *α* increases in magnitude due in part to the spin de-coherence at higher *t*_*Ta*_ and also in part due to decrease of Rasbha like spin orbit torque away from interface. The observed decrease in effective Gilbert damping constant in *β*-Ta/Py bilayer is very promising, and demonstrates the potential of using Ta based nanostructures in developing low power spintronic devices as it no more necessitates the presence of DC-current for tuning the damping parameter.

## Additional Information

**How to cite this article**: Behera, N. *et al.* Anomalous anti-damping in sputtered *β*-Ta/Py bilayer system. *Sci. Rep.* **6**, 19488; doi: 10.1038/srep19488 (2016).

## Figures and Tables

**Figure 1:**
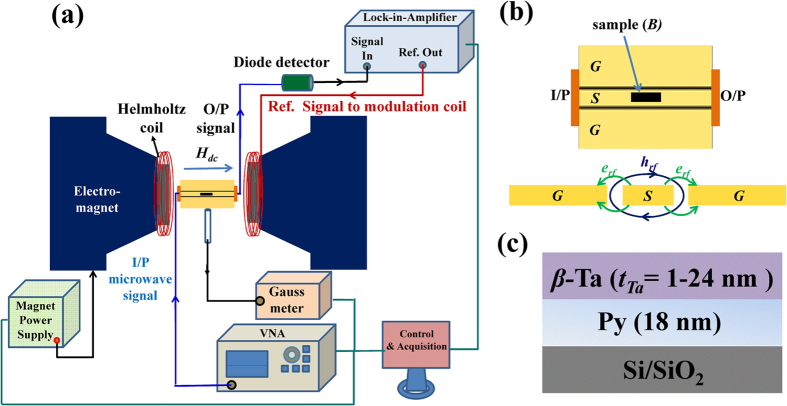
(**a**) Schematic diagram of the LIA based FMR measurement system. (**b**) Schematic of CPW on which bilayer sample *B* is kept in contact with the central signal transmission line (*S*) which is isolated from the adjacent ground lines (*G*). I/P and O/P represent the input and output signal ports of CPW. Also shown is the microwave field (*h*_*rf*_ and *e*_*rf*_) distribution inside the CPW. (**c**) Sample configuration (size ~1 × 4 mm^2^) showing the various layers.

**Figure 2:**
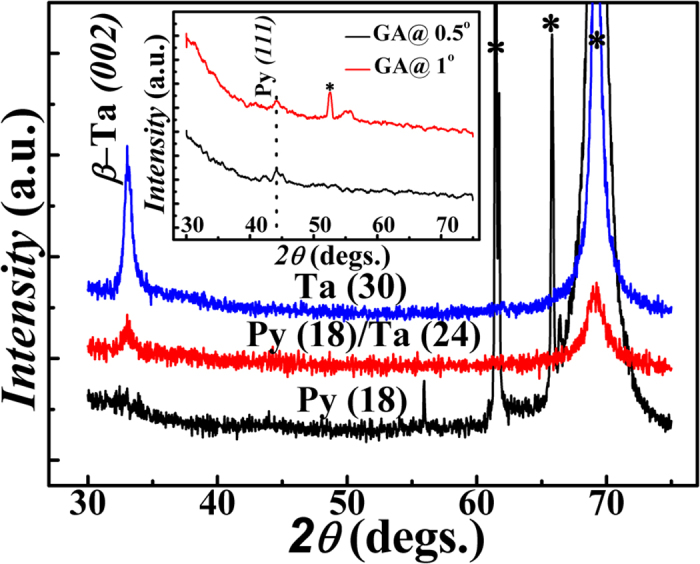
XRD patterns of Py(18 nm), *β*-Ta(30 nm), *β*-Ta(24 nm)/Py(18 nm), films. It may be noted that the *(004)* peak of *β*-Ta which occurred at 70.1° merged with the *(004)* peak of Si. figure shows the glancing angle XRD patterns of bare Py(18 nm) thin film at glancing angle θ corresponding to 0.5° and 1°, respectively. The peaks marked with star (*) symbols correspond to the peaks from Si substrate.

**Figure 3:**
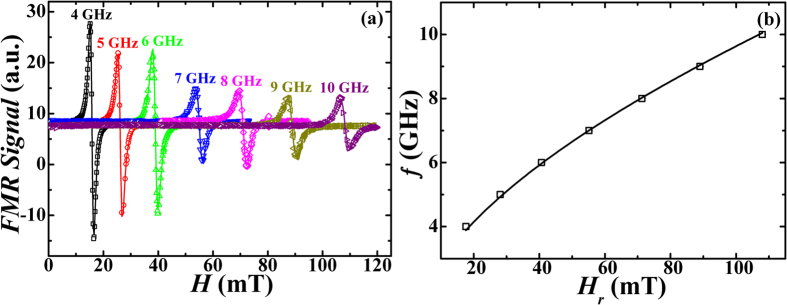
(**a**) FMR spectra recorded at different indicated constant microwave frequencies for *β*-Ta(1 nm)/Py(18 nm) thin film. The symbols represent experimental data while lines are the fits. (**b**) The corresponding *f* vs *H*_*r*_ plot. Open symbols are experimental data while the solid line is a fit using Kittel’s formula, equation (2).

**Figure 4:**
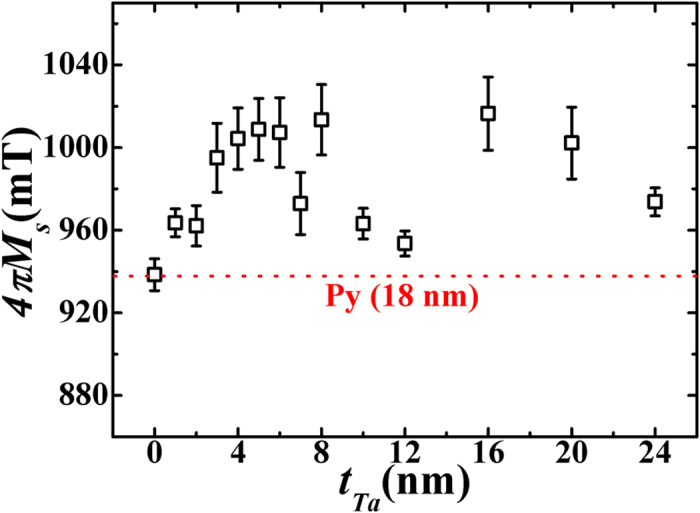
Variation of saturation magnetization ***4πM***_***s***_ **of the** ***β*****-Ta (*****tT***_***a***_**)/Py(18 nm) bilayer thin films obtained from the fittings of equation** (2) . Square symbols are the experimental data which are shown together with error in estimation. The dashed line represents the *4πM*_*s*_ value as measured from the FMR measurement corresponding to a bare Py film.

**Figure 5:**
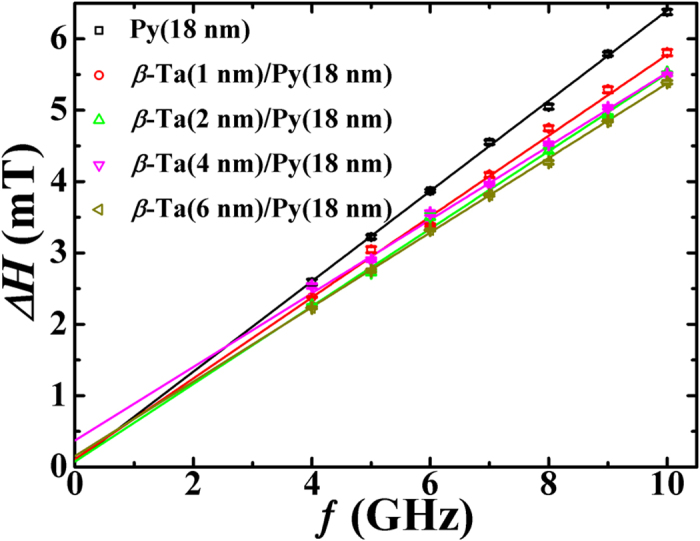
Frequency (*f*) versus linewidth (Δ*H)* for different thicknesses of *β*-Ta layer over constant Py(18 nm). Open symbols are experimental data while the solid lines are fits with equation (3).

**Figure 6:**
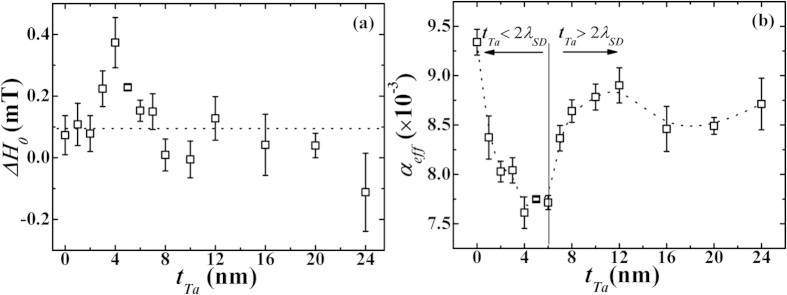
Variation of (**a**) inhomogeneous broadening (Δ*H*_*0*_) (**b**) effective Gilbert damping constant 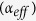 as a function of 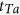 for *β*-Ta(*t*_*Ta*_)/Py(18 nm) bilayers. Both dashed and solid lines are guide to the eye.

**Figure 7:**
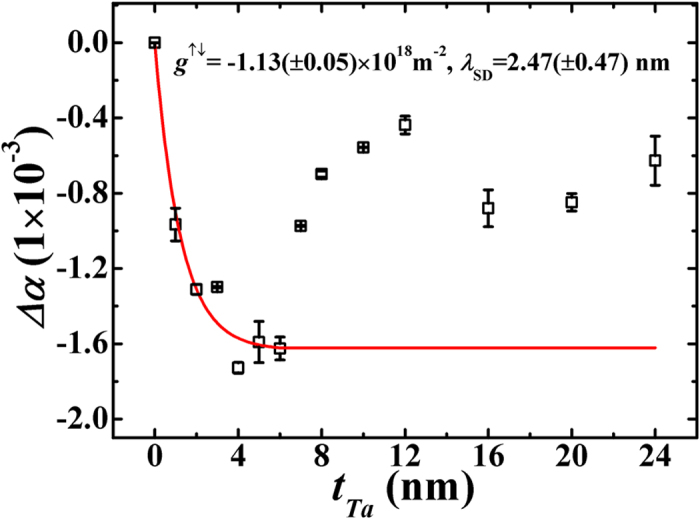
Δ*α*vs. *t*_*Ta*_ for *β*-Ta(*t*_Ta_)/Py(18 nm). Open symbols are experimental data while the red solid line is a fit with equation (5).

**Figure 8:**
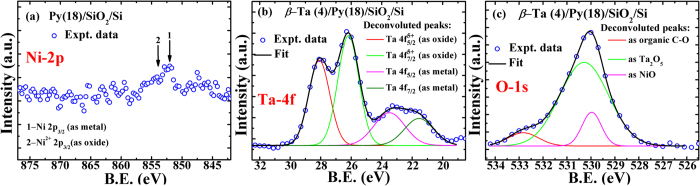
XPS spectra corresponding to Ni-2p (**a**) Ta-4f (**b**) and O-1s (**c**) levels recorded on the nominal Py(18 nm) and *β-*Ta(4 nm)/Py(18 nm) bilayer. Symbols represent experimental data. Lines represent the indicated component fits.

**Figure 9:**
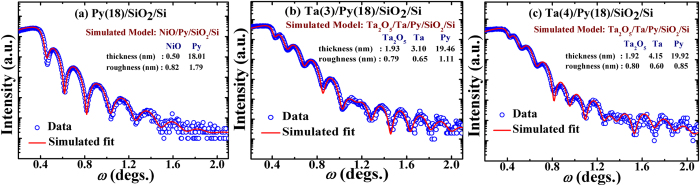
X-ray reflectivity data and simulated profiles for *β*-Ta(*t*_*Ta*_)/Py(18 nm)/SiO_2_/Si samples having different *t*_*Ta*_, (**a**) 0 (**b**) 3 nm, and (**c**) 4 nm. Also shown in the panel are the fitted values of the thickness and roughness of the individual modeled layers with errors in the range of 0.05–0.10 nm.
